# Stigmatization and resilience in inflammatory bowel disease patients at one-year follow-up

**DOI:** 10.3389/fgstr.2022.1063325

**Published:** 2022-12-15

**Authors:** Marco Vincenzo Lenti, Giacomo Broglio, Caterina Mengoli, Sara Cococcia, Federica Borrelli de Andreis, Marta Vernero, Lavinia Pitotti, Lucia Padovini, Matteo Secco, Mariangela Delliponti, Gino Roberto Corazza, Catherine Klersy, Antonio Di Sabatino

**Affiliations:** ^1^ Department of Internal Medicine and Medical Therapeutics, University of Pavia, Pavia, Italy; ^2^ First Department of Internal Medicine, Fondazione Istituto di Ricerca e Cura a Carattere Scientifico (IRCCS) Policlinico San Matteo, Pavia, Italy; ^3^ Clinical Epidemiology and Biometry, Fondazione Istituto di Ricerca e Cura a Carattere Scientifico (IRCCS) Policlinico San Matteo, Pavia, Italy

**Keywords:** Connor-Davidson resilience scale, Crohn’s disease, inflammatory bowel disease, stigma, self-efficacy, self-esteem

## Abstract

**Introduction:**

Inflammatory bowel disease (IBD), namely ulcerative colitis and Crohn’s disease, is a chronic relapsing immune-mediated condition that may cause an impairment of social functions due to stigmatisation. Resilience instead is associated with an improvement in coping with adversities and thus may counteract the detrimental effects of stigmatisation. We herein sought to determine the fluctuation of stigmatisation and resilience in a cohort of patients with IBD at 1-year follow-up.

**Methods:**

This is a prospective, monocentric study conducted in a tertiary referral centre. All patients with IBD were assessed at enrolment and at oneyear follow-up. Several clinical and demographic variables were collected. Stigmatisation was assessed through a validated Italian version of the Perceived Stigma Scale for IBD (PSS-IBD), while resilience was assessed through the 25-item Connor Davidson Resilience Scale (CD-RISC25). Also, self-efficacy (SEF) and self-esteem (SES) scales were assessed.

**Results:**

In this study, 105 patients were included (46 Crohn’s disease, 59 ulcerative colitis; overall mean age 47 years ±11, M:F ratio 1:1.2). None of the 4 scales showed a statistically significant variation at one year compared to baseline (median CD-RISC25 64 at baseline vs 61 at follow-up; SEF 31 vs 30; SES 32.5 vs 32; PSS-IBD 0.45 vs 0.45). A statistically significant and inverse correlation was found between CD-RISC25 and PSS-IBD (rho -0.222, p=0.01), SEF and PSS-IBD (rho -0.219, p= 0.01), SES and PSS-IBD (-0.316, p=0.003). CD-RISC25 was found to be positively associated with inactive IBD (p=0.05).

**Discussion:**

In this prospective study we have shown for the first time that stigmatisation, resilience, SEF and SEM did not change over a one-year time span, suggesting that, based on the information gathered, these characteristics may be independent from IBD severity or IBD flares. Furthermore, we found an inverse correlation of stigma with resilience, SEF and SES, suggesting an important role that these variables may have on preventing stigmatisation.

## Introduction

Inflammatory bowel disease (IBD), namely Crohn’s disease (CD), ulcerative colitis (UC), and IBD unclassified (1), is an immune-mediated chronic condition characterized by periods of relapse and remission (2), that has a deep impact on the patients’ health, quality of life, and psychological dimension. This is the consequence of a) pervasive symptoms (3) experienced by patients with IBD, such as chronic abdominal pain, diarrhoea, rectal bleeding, and fatigue; b) the risk of receiving surgical treatment (4) and; c) the marked impact of IBD on sexual life (4). Another cause of psychological burden in patients with IBD is social stigma (5, 6) which is defined as the feeling or fear that other people may have a negative attitude towards someone due to specific attributes (6–8), leading to a loss of *status quo* and to discrimination (7). In a clinical setting, stigma can be categorized into perceived stigma (when a negative attitude is felt by an individual), enacted stigma (discriminating acts), and internalized, self-stigma (8). Stigma is known to have a detrimental impact on several chronic diseases, such as psychiatric disorders (9), HIV (10, 11), and epilepsy (12). Stigma may be experienced by up to 84% of patients with IBD, regardless of disease activity (13); moreover, stigma is also associated with a higher prevalence of depression, social withdrawal, and poorer quality of life (14–16). Stigmatisation can be measured in patients with BID with a scale adapted from patients with irritable bowel syndrome (IBS), known as Perceived Stigma Scale (PSS) (17, 18).

Resilience is a positive psychological resource that may potentially counteract the detrimental effects of stigma on quality of life (19). Resilience can be found in three main dimensions, namely personality traits (the innate ability to address negative situations), outcomes (the positive impact that resilience has on the disease’s impact), and processes (the dynamic process, namely coping with a chronic condition) (20). Interestingly, resilience in IBD has been found to be associated with a more favourable outcome (21–23), and it is linked to some individual characteristics, including age, sex, employment status (24, 25). Additionally, it can be trained *via* resilience-enhancing programs and the development of mindfulness (26–28). Resilience can be measured with objective scales, and one of the most used worldwide is the Connor-Davidson Resilience Scale (CD-RISC25) (29).

Finally, self-efficacy (SEF) and self-esteem (SES) may also play an important role in relation to stigma and resilience. SEF is defined as the confidence to independently manage the disease without needing a caregiver (30) and it has been found to favourably affect the outcome of chronic diseases (31), though being a task-specific skill (31–33) and therefore a non-transferable skill from other aspects of life; furthermore, self-efficacy can predict health promoting behaviour in chronic ill patients, regardless of disease activity and severity (34). SES instead plays an important role in mental health, as a lack of it has been linked to a higher rate of depression and anxiety (35, 36); notably, in the IBD setting, it can be measured by a scale developed by Rosenberg et al. (36, 37).

Because of limited data on stigma in IBD and because of the absence of a validated scale for PSS-IBD in the Italian language, we previously performed a study for validating an Italian version of the PSS-IBD (29). By using a three-step method, the validation showed acceptable translation and psychometric properties, with an excellent item internal consistency (Cronbach alpha 0.87). Additionally, we assessed stigmatisation, resilience, SEF, and SES at baseline; we found that resilience negatively correlated with perceived stigma. In the present paper we report the one-year follow-up results of the aforementioned prospective study (29), as well as other important outcomes, including the associations between stigma, resilience, SEF, and SES in relation to IBD activity.

## Material and methods

### Study population

IBD patients followed-up at the IBD Clinical & Research Centre of the San Matteo Hospital Foundation were consecutively enrolled between December 2018 and September 2019 and we originally planned to follow them up after 12 months. However, due to the occurrence of the SARS-CoV-2 pandemic, the follow-up period was extended until July 2021. The initial sample consisted of 126 IBD patients, though 24 dropped out at the follow-up evaluation. Briefly, all IBD diagnoses followed the international guidelines (1, 38). Patients were eligible for inclusion if they had at least a 3-month history of IBD, were aged ≥18, were able to complete a questionnaire, and were willing to provide written informed consent; patients with an inconclusive or uncertain diagnosis of IBD, those diagnosed less than 3 months before or unwilling to provide informed consent were excluded. Demographic and clinical characteristics were gathered, including IBD type, disease activity and duration, comorbidities, and previous IBD-related surgery.

### Disease activity

We evaluated disease activity in CD with the Harvey-Bradshaw index (HBI) (39), while using the Partial Mayo Index for UC (40). HBI is a score that considers many factors, spacing from general well-being, abdominal pain, number of liquid or soft stools, the presence of abdominal mass and complications, if any (namely arthralgia, uveitis, erythema nodosum and others). An HBI of <5 defines disease remission, an HBI of 5-7 a mild disease, an HBI of 8-16 a moderate disease, and an HBI of >16 a severe disease. The Partial Mayo Score evaluates stool frequency, rectal bleeding, and the physician rating of disease activity. A Partial Mayo Score of <2 defines disease remission, a score of 2-4 a mild disease severity, a score of 5-7 a moderate disease severity, and a score of >7 a severe disease severity. By active disease, we considered either a Partial Mayo Score of ≥5 for UC or an HBI of ≥ 8 for CD.

### Stigma

Stigma was assessed by using the Italian validated version of the PSS-IBD (41) scale, a self-administered questionnaire composed of 10 items and scoring the perceived stigma from 0 (never) to 4 (always) points, with the higher ranking meaning a higher level of stigma. The score was performed both for the patient’s “significant others” (SO) (i.e., the social background, family, friends) and for the “healthcare professional” (HP), leading to a total of 20 overall evaluated items. Both evaluations are important, as are part of the original PSS-IBS. The distinction between SO and HP was deemed important as patients with IBS may be more likely to be considered as having a psychosomatic disorder, thus increasing stigma. Also, measuring the perceived stigma from patients is pivotal because it can have an impact on the patients’ trust on HP. The final stigma score was then obtained by calculating all the values and mean of all values of the items (SO+HP).

### Resilience

Resilience was evaluated using the Italian-translated version of the Connor-Davidson Resilience Scale (CD-RISC25) (29) which consists of a self-administered questionnaire composed of 25 items on a five-point Likert scale, where 0 stands for strongly disagree and 4 for strongly agree, and the final score is obtained with the sum of all the individual scores, with a higher score underlying a higher resilience (42).

### Self-esteem

The Rosenberg SES scale, is a 10-item scale. Scoring involves combined ratings from 1 to 4 points; low SES answers are “disagree” or “strongly disagree” on items 1, 3, 4, 7, 10, and, conversely, “strongly agree” or “agree” on items 2, 5, 6, 8, 9 (35, 36, 43) with higher scores underlying a higher level of SES.

### Self-efficacy

SEF is the ability to cope with the needs and the medical adherence in a chronic disease. In IBD this skill was measured with a scale developed by Keefer et al. in 2011 (41) and it consists in an interview with 29 items that explore various disease-related areas with item scores varying from 1 (not at all) to 10 (totally). The 4 areas that were evaluated were as floow: managing stress and emotions, managing medical care, managing symptoms and diseases, maintaining remission (44), with the higher the score, the higher the SEF.

### Statistical analysis

We used the Stata software (release 17, StataCorp, College Station, TX, USA) for computations. A 2-sided p-value <0.05 was considered as statistically significant. We described continuous variables with the mean and standard deviation (SD) or the median and quartiles (IQR), if skewed; we described categorical variables as counts and percent. We used the signed rank test for comparisons of questionnaire scores and the exact McNemar test to compare disease activity over time. We used the Mann Whitney U test to compare scores between patients with active or inactive disease at time 0; we used the Spearman R and 95% confidence interval (95% CI) to measure the correlation of scores at time 0. The study was approved by the local Ethics Committee (Protocol #20190003611), and all participants gave their informed written consent to take part to the study and for the anonymized publication of data.

## Results

In our study, 105 patients of the previously 125 enrolled (84%) completed the follow-up. The demographic and clinical characteristics are summarized in **Table 1**. Of the overall 105 patients, 45.2% had Crohn’s disease and 54.8% had ulcerative colitis. Regarding therapy, 14 (13.5%) of patients were under biological therapy, with infliximab being the most prescribed (7 patients; 50%) and 38 (36%) were given 5-aminosalycilic drugs. Finally, five (4%) patients were not taking medications.

**Table 1:** Demographic and clinical characteristics of the cohort under study.

	Overall (105)	CD (46)	UC (59)	p-value
Age (mean ±*SD*)	47 (±16.93)	44 (±15.91)	50 (±17.15)	
Male	59	27	32	
BMI	24.42(±4.2)	24.42 (±.3.8)	24.42(±4.4)	0.39
Disease Duration (median. IQR) – years	13.68 (11.55-15.72)	13.45 (6.73-26.91)	10.56 (5.27-21.11)	
Disease Characteristics				
Location (CD)				
Terminal Ileum (L1)		11 (23.9%)		
Colon (L2)		4 (8.7%)		
Ileo-colon (L3)		31 (67.4%)		
Upper GI (L4)		/		
Perianal Disease (p)		15 (32.6%)		
Behaviour (CD)				
Inflammatory (B1)		16 (34.7%)		
Stricturing (B2)		25 (54.3%)		
Penetrating (B3)		15 (32.6%)		
Disease Activity (HBI)				
<5		80.0%		0.78
5-7		15.6%		0.75
8-16		4.4%		0.21
Disease Characteristics (UC)				
Location				
Proctitis (E1)			4 (6.8%)	
Left-sided (E2)			21 (35.6%)	
Extensive (E3)			34 (57.6%)	
Disease Activity (pMayo)				
<2			69.5%	0.67
2-4			27.1%	0.66
5-7			1.7%	0.88
>7			1.7%	0.82
Pouch			2 (3.4%)	
Extraintestinal Manifestations	30 (28.5%)			
Previous Abdominal Surgery	25 (26.2%)			
Calprotectin				
<50	38.4%			0.25
51-250	42.4%			0.9
>250	19.2%			0.6
CRP				
Normal	76.5%			0.8
Raised	10.2%			0.7
Missing	13.3%			0.9
Comorbidities				
Cardiopathy	14.3%			
Arterial Hypertension	24.7%			
Diabetes	8.7%			
Hepatic failure	0.8%			
Respiratory failure	3.2%			
Renal failure	1.6%			
Neurological diseases	3.9%			
Onco-haematological diseases	8.7%			
Psychiatric disorders of which	11.9%			
Anxiety	7.1%			
Depression	6.3%			
Others	0.8%			

### First end-point

At one-year follow-up, 4 questionnaires were assessed, namely CD-RISC 25, PSS, SES and SEF scales (**Table 2**). No statistically significant difference was found between baseline and follow-up for all questionnaires. The CD-RISC25 values were 64 at baseline (IQR 54-79 and 61 at follow-up (IQR 51-73; p=0.12); the SEF values were 31 at baseline (IQR 28-34) and 30 at follow-up (IQR 27-33; p=0.12); the SES values were 32.5 at baseline (IQR 28-34) and 32 at follow-up (IQR 29-34,5; p=0.17). We also evaluated the PSS-IBD scale that showed higher values for the significant others (PSS-SO) stigma versus the healthcare (PSS-HP) one, with no statistically significant difference between baseline and follow-up. PSS-SO was 0.7 (IQR 0.4-1.4) at baseline and 0.7 (IQR 0.4-1.3) at follow-up (p=0.54); PSS-HP was 0.1 both at baseline and at follow-up (p=0.96) and PSS-SUM, obtained by adding the scores of HP and SO, was 0.45 at baseline (IQR 0.25-0.9) and 0.45 (IQR 0.2-0.85) at follow-up (p=0.8).

**Table 2:** Baseline vs follow-up values of the questionnaires assessed in the study.

	CD-RISC25	PSS	SES	SEF
**Baseline**	64 (IQR 54-78)	31 (IQR 28-34)	32.5 (IQR 28-34)	31 (IQR 28-34)
**Follow-up**	61 (IQR 51-73)	30 (IQR 27-33)	32 (IQR 29-34.5)	30 (IQR 27-33)
**p-value**	p= 0.12	p=0.12	p=0.17	p=0.12

### Second end-point

Correlations among the various questionnaires were assessed *via* the Spearman’s Rho (**Table 3**). Notably, an inverse correlation between PSS (and its subgroups) and CD-RISC25 was noticed in almost all cases, as well as for SES or SEF and PSS (and its subgroups).

**Table 3:** Correlations between SES, SEF, CD-RISC25 and PSS.

	Spearman’s Rho		95% CI		p-value	
SES – PSS SO	-0.3089		-0.460/ -0.140		0.005	
SES – PSS HP	-0.2226		-0.384/ -0.048		0.013	
SES – PSS SUM	-0.3166		-0.467/ -0.149		0.0003	
SEF – PSS SO	-0.2254		-0.386/ -0.51		0.012	
SEF – PSS HP	-0.1731		-0.339/ -0.003		0.055	
SEF – PSS SUM	-0.2192		-0.381/ -0.045		0.014	
CD-RISC25 – PSS – SO	-0.2217		-0.382/-0.048		0.012	
CD-RISC25 – PSS – HP	-0.1649		-0.331/0.011		0.06	
CD-RISC25 – PSS – SUM	-0.2221		-0.383/ -0.048		0.013	

### Third end-point

Lastly, we evaluated disease activity and its correlation with CD-RISC25 and PSS, and the effect that reaching disease remission at one year could have on these two variables (**Table 4**). Of the 126 patients, at baseline 86% were in remission and 14% (n=15) had an active disease (Partial Mayo Score ≥5 for UC or HBI ≥8 for CD). Disease remission was defined as for all the patients who had active disease on baseline and had partial mayo scores <5 and HBI <8 at follow-up. Of note, CD-RISC25 was inversely and significantly associated with disease activity. Disease remission at follow-up was achieved in 14 (92%) patients who had active disease at baseline, and this had no impact on both stigma and resilience.

**Table 4:** Correlation between disease activity and questionnaire scores, and between those who reached disease remission at one-year follow-up and questionnaire scores.

Patients with active disease [n=15 (14%)]	Median (IQR)	z value	P-value
**CD-RISC25**	59 (43-69)	1.95	0.05
**PSS-SO**	0.90 (0.20-1.90)	-1.08	0.27
**PSS-HP**	0.10 (0-0.80)	-0.856	0.39
**PSS-SUM**	0.45 (0.35-1.50)	-1.08	0.27
Patients who achieved disease remission at one-year follow-up [n=14 (12.8%)]			
**CD-RISC25**	59 (46.5-76)	-0.35	0.72
**PSS-SO**	0.85 (0.15-1.65)	1.19	0.23
**PSS-HP**	0.20 (0.10-0.45)	0.87	0.15
**PSS-SUM**	0.55 (0.13-1.02)	0.68	0.53

## Discussion

In this prospective study we have shown for the first time that stigmatisation, resilience, SEF and SEM did not change over a one-year time span, suggesting that, based on the information gathered, these characteristics may be independent from IBD severity or IBD flares. Furthermore, we found an inverse correlation of stigma with resilience, SEF and SES, suggesting an important role that these variables may have on preventing stigmatisation.

The first endpoint of our study consisted in the follow-up evaluation of the CD-RISC25, the PSS-IBD, and the SEF and SES scales in order to assess how they changed over time. Interestingly, for all of these scales, no statistical difference emerged, suggesting that stigma and resilience are stable over time in IBD, at least over one year, differently to what is seen for IBS (35, 37). Furthermore, a strong “floor effect” was evident with regard to stigmatisation; this was, in fact, particularly low, especially the HP sub-score, probably because these patients were accessing a tertiary referral centre for IBD management, with well-trained doctors and nurses having a long lasting experience, as we postulated in our previous paper (29). Even considering this explanation, the levels of stigmatisation experienced by our patients were even lower than those reported in a similar, previously published, paper (18). We may speculate that the patients included in our study had, on average, a longstanding disease, and hence they had more time to cope and adapt to IBD. There may also be other factors, namely cultural and behavioural, that were not specifically addressed in this study. To note, the follow-up period coincided with the outbreak of the COVID-19 pandemic that may have played an independent role in determining stigma and resilience levels.

Interestingly, what emerged from our second endpoint was that stigma and resilience have an inverse correlation, as well as SES and SEF, with regard to stigma. We noted that CD-RISC25 was inversely related to the PSS-SO, PSS-HP (although at the limit of significance), and PSS-SUM. Stigma, SEF and SES had a similar trend. This suggests that interventions aimed at improving resilience, SES, and SEF may potentially counteract the perceived stigmatisation; therefore, interventional studies are needed to clarify this topic, as there is no available evidence.

Finally, in the third endpoint of our study we noted that resilience was inversely associated with an active disease, while stigma was inversely correlated with disease activity, although not significantly. Lastly, reaching disease remission at one year did not have any statistically significant correlation with the CD-RISC25 or the PSS-IBD. These results may be related to the small sample size of patients with an active disease who reached remission in our cohort. Nonetheless, various results can be found in previously published papers (13, 14, 29) in this regard, although with a small sample size. In our previously published paper, only for UC patients, disease activity correlated with higher PSS-SO, while no other associations among PSS and disease activity in CD were noticed (29). In another study, disease activity was found to worsen quality of life, but a formal correlation between stigma and disease activity was not made (13). Finally, in a study looking at stigma and depressive symptoms in young IBD patients, there were too few patients with an active disease for drawing firm conclusions (14). Hence, further larger studies are needed to better address this issue.

Our study certainly has some limits. First, our cohort had a consistent floor effect both on perceived stigma and disease activity, and the follow-up period was affected by the COVID-19 outbreak. The sample size was relatively small, therefore, further studies with larger cohorts are needed to better assess these topics. Our study has some strengths as well, as it was the first prospective, clinical study that evaluated how stigma and resilience changed over time in in an Italian cohort of IBD patients, demonstrating not only that these two characteristics are stable over one year, but even that SEF and SES are stable as well. Furthermore, we noticed that stigma is inversely correlated with resilience, SES and SEF, and this finding paves the way for a more holistic approach that should include a psychological assessment and support in IBD patients.

## Conclusions

IBD patients are complex patients who may be burdened by psychological and physical issues that need to be recognised and correctly assessed by their treating physicians, in order to offer to these patients a higher quality of life. A more holistically-driven approach, with the intervention of a multidisciplinary team may help bringing a special focus on reducing stigma and improving resilience, SEF, and SES in this population. Other factors potentially affecting stigma and resilience in IBD should be investigated.

## Data availability statement

The original contributions presented in the study are included in the article/supplementary material. Further inquiries can be directed to the corresponding author.

## Ethics statement

The studies involving human participants were reviewed and approved by San Matteo Hospital Foundation. The patients/participants provided their written informed consent to participate in this study.

## Author contributions

All authors participated in the drafting of the manuscript or critical revision of the manuscript for important intellectual content and provided approval of the final submitted version. All authors contributed to collect data, writing the manuscript, and reviewing the paper.
